# Supplementary material for: Distinct Molecular Profiles Underpin Mild-To-Moderate Equine Asthma Cytological Profiles
Source: Cells. 2024 Nov 20;13(22):1926. doi: 10.3390/cells13221926 (PMC11593015; doi:10.3390/cells13221926)
Supplement: Supplementary file 1 [file cells-13-01926-s001.zip › Supplementary_Material S1qPCR Results.pdf]

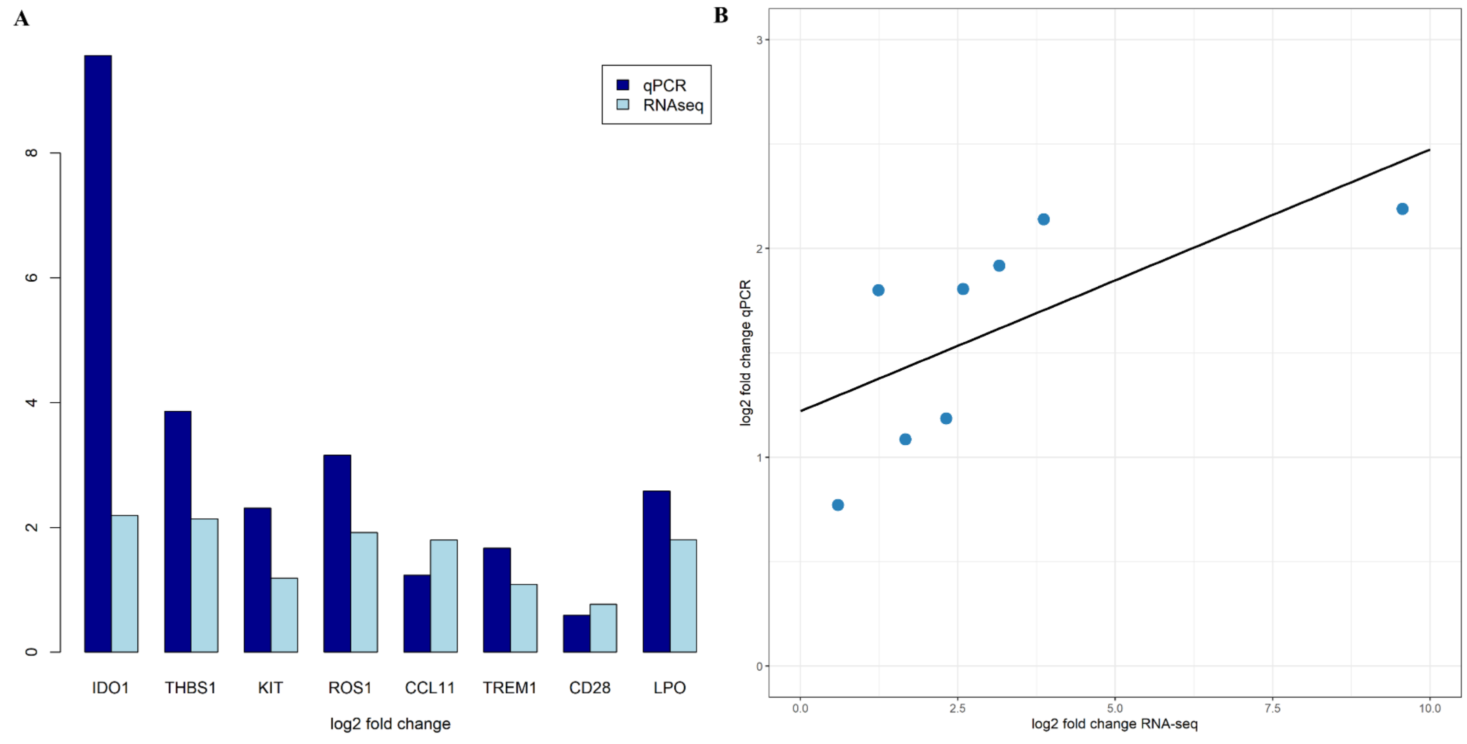

Expression profile of RNA-seq and qPCR data. *Expression profile of RNA-seq and qPCR data for selected genes across all comparisons were compared using the same samples.* ***A)*** *The data are consistent with the results of RNA-seq analysis for these genes in the same samples; IDO1, THBS1 (Group_B_NEUT), KIT, ROS1 (Group_C_MAST), CCL11, TREM1 (Group_D_NEUT_MAST), CD28, LPO (Group_E_EOS_MAST). Expression was normalized using SDHA as a reference gene in qPCR. Results represent mean values and are expressed as log2 values of the fold change and are representative of a minimum of 3 biological replicates.* ***B)*** *RNA-seq and qPCR log2 values of the fold change were significantly correlated with a correlation coefficient r = 0.93 (Spearman's rank correlation test) and P value = 0.002), after testing for normality using shapiro.test. Statistical analysis and graphs were generated in R.*

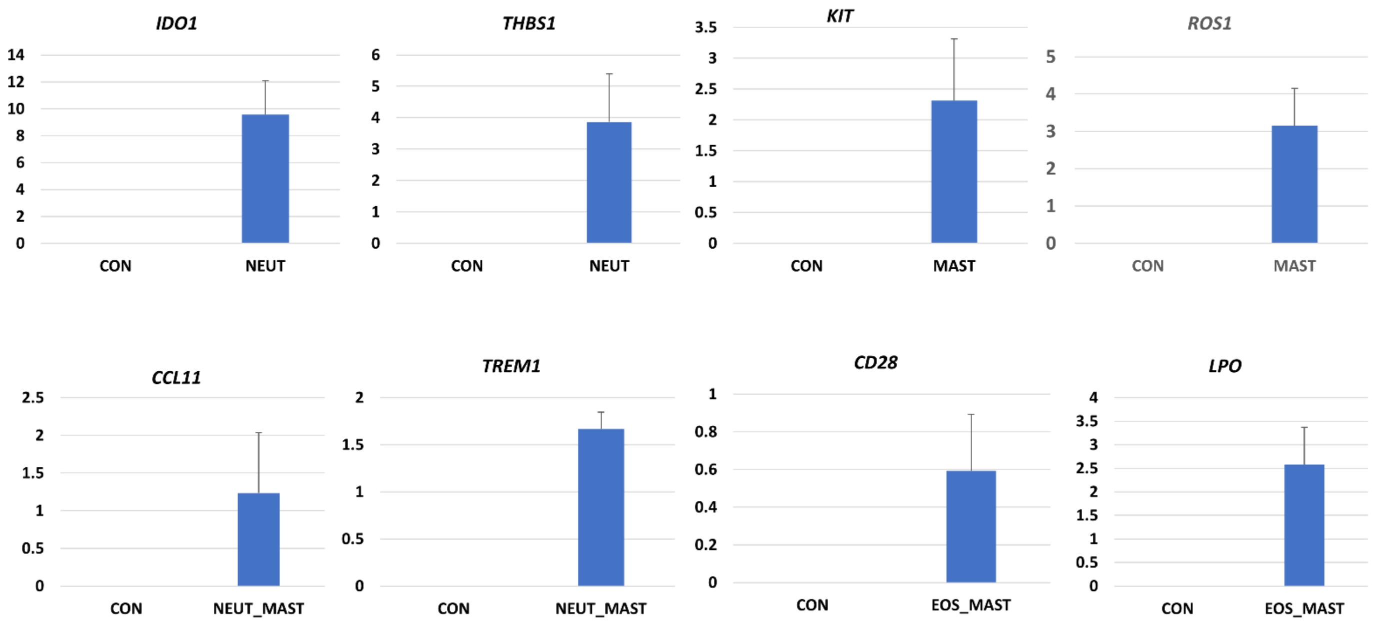

Expression profile of qPCR data. *The relative gene expression of selected genes, obtained from qPCR data, is presented across all comparisons. qPCR was performed on the same samples used for the RNA-seq analysis. IDO1, THBS1 were tested in Group_B_NEUT* versus control, *KIT, ROS1 in Group_C_MAST versus Control, CCL11, TREM1 in Group_D_NEUT_MAST versus Control, and CD28, LPO in Group_E_EOS_MAST versus Control. Expression was normalized using SDHA as a reference gene in qPCR. Results represent mean values and are expressed as log2 values of the fold change and are representative of a minimum of 3 biological replicates.*

Primers used in this study

Target	Primer orientation	Primer Sequence (5’-3’)	Size (bp)
IDO1	F	ATCAAAGAAATTCCGTTATATTCAA	94
	R	TGCGTAGACAAGAAGAAGTTATATCAAT	
THBS1	F	CAGACTCCGCATCGCAAAAG	134
	R	GGAGGACATTGGTAGAGCTGG	
KIT	F	AGAGCTGTGCCAGTTGTGTC	137
	R	CTGTTCTTTAGTCCGGCTGT	
ROS1	F	ATCTGAGTGAACCGTGCATCC	71
	R	TTCCTCTTCGTACGCACCTT	
CCL11	F	ACCAGCCCAGAAACCAACAG	102
	R	ACGGTCGAGATAGAAACTGGCT	
CD28	F	TCTTTGTACTTGCTGGATGAGG	110
	R	CGTAGGGCTGGTAGTGCTTTC	
LPO	F	CGCTTTTTGGAAGGACCATGT	124
	R	AGACAGACGTGCGAGGAAAC	
SDHA	F	GGACAGAGCCTCAAGTTTGG	116
	R	GTATCATATCGTAGAGACCTTCCATA	
